# Predicting Atrial Fibrillation Risk in Hypertrophic Cardiomyopathy Patients: Left Atrial Phasic Function and Strain Mechanics

**DOI:** 10.1016/j.cjco.2026.04.005

**Published:** 2026-04-24

**Authors:** Mahdi Montazeri, Negin Rashidi, Deacon Z.J. Lee, Ali Sakhnini, Sara Hoss, Arnon Adler, Harry Rakowski, Raymond H. Chan

**Affiliations:** a Division of Cardiology, Toronto General Hospital, Toronto, Ontario, Canada

**Keywords:** atrial fibrillation, hypertrophic cardiomyopathy, left atrial strain, booster function, CMR

## Abstract

**Background:**

To evaluate whether the left atrium (LA) phasic function and myocardial deformation by cardiovascular magnetic resonance (CMR) in hypertrophic cardiomyopathy (HCM) patients is a predictor of atrial fibrillation (AF) development.

**Methods:**

The baseline CMRs of HCM patients who later developed AF (n = 140; mean age = 61 ± 12 years; male = 86) were compared with those of HCM patients who did not develop AF (n = 70; mean ag e= 58 ± 11 years; male patients = 48). This case–control study was conducted at our quaternary referral centre between January 2001 and June 2021. The univariate and multivariable Cox proportional hazards model was used to assess the correlation between clinical and imaging parameters vs the likelihood of developing AF.

**Results:**

Compared to the non-AF cohort, HCM patients with AF had larger baseline LA volumes and lower LA strain and LA strain rates. Maximum left ventricular (LV) wall thickness, absolute and indexed LV mass, and indexed LV end-diastolic volume were larger in patients with AF vs the non-AF group. LV ejection fraction assessed by CMR along with late gadolinium enhancement prevalence and pattern was similar between patients with vs without AF.

LA booster strain (5.8 ± 3.4 in the AF group vs 11.4 ± 4.2 in the non-AF group, *P* < 0.00001) and total LA ejection fraction (32.9 ± 12.8 in the AF group vs 44.0 ± 9.3 in the non-AF group, *P* < 0.00001) were significantly worse at baseline in the AF group. No significant relationship was present between markers of LV disease burden and AF.

**Conclusions:**

LA booster strain and total LA ejection fraction at baseline CMR correlated with AF development in HCM patients.

## Résumé

**Contexte:**

Évaluer si la fonction phasique de l'oreillette gauche (OG) et la déformation myocardique, telles qu'elles sont mesurées par résonance magnétique cardiovasculaire (RMC) chez les patients atteints de cardiomyopathie hypertrophique (CMH), constituent un facteur prédictif de l'apparition d'une fibrillation auriculaire (FA).

**Méthodologie:**

Les données de RMC initiales de patients atteints de CMH ayant ultérieurement développé une FA (n = 140; âge moyen = 61 ± 12 ans; hommes = 86) ont été comparées à celles de patients atteints de CMH n'ayant pas développé de FA (n = 70; âge moyen = 58 ± 11 ans; hommes = 48). Cette étude cas-témoins a été menée dans notre centre de référence quaternaire entre janvier 2001 et juin 2021. Le modèle de Cox à risques proportionnels, univariés et multivariés, a été utilisé pour évaluer la corrélation entre les paramètres cliniques et d'imagerie et la probabilité de développer une FA.

**Résultats:**

Par rapport à la cohorte sans FA, les patients atteints de CMH avec FA présentaient des volumes de l'oreillette gauche (OG) initiaux plus importants et une déformation ainsi que des vitesses de déformation de l'OG plus faibles. L'épaisseur maximale de la paroi ventriculaire gauche (VG), la masse VG absolue et indexée, ainsi que le volume télédiastolique indexé du VG étaient plus élevés chez les patients atteints de FA que chez ceux sans FA. La fraction d'éjection ventriculaire gauche (FEVG) évaluée par RMC, ainsi que la prévalence et le profil du rehaussement tardif au gadolinium, étaient similaires entre les patients avec et sans FA.

La déformation de contraction active de l'OG (5,8 ± 3,4 dans le groupe FA vs 11,4 ± 4,2 dans le groupe sans FA, *p* < 0,00001) et la fraction d'éjection de l'OG totale (32,9 ± 12,8 dans le groupe FA vs 44,0 ± 9,3 dans le groupe sans FA, *p* < 0,00001) étaient significativement plus faibles au départ dans le groupe FA. Il n'y avait pas de relation significative entre les marqueurs de la charge pathologique du VG et la FA.

**Conclusions:**

La déformation de contraction active de l'OG et la fraction d'éjection de l'OG totale initiale de la RMC étaient corrélées au développement de la FA chez les patients atteints de CMH.

Hypertrophic cardiomyopathy (HCM) is a primary myocardial disease characterized by otherwise unexplained left ventricular hypertrophy, typically with asymmetric septal involvement. HCM affects one in every 500 adults, and atrial fibrillation (AF) is the most common sustained arrhythmia among HCM patients, with a prevalence 4-6 times higher than that in the general population.^1^ HCM patients with AF are at higher risk of thromboembolic events, functional capacity decline, and heart failure–related mortality.^2^^,^^3^

The pathophysiological basis for this vulnerability is not fully understood, although atrial arrhythmogenic remodelling seems to play an important role.^4^ Previous predictive models assessing AF in the general population have not been validated in HCM cohorts. Recently, Carrick et al. presented the Hypertrophic Cardiomyopathy–Atrial Fibrillation score (HCM-AF) score to assess AF risk in HCM individuals, which included age at clinical assessment, age at HCM diagnosis, left atrial (LA) diameter and heart failure symptoms.^5^ In addition, a number of clinical and imaging features linked to AF have been investigated in small studies and cohorts, including include LA size, age, p-wave duration, baseline ST-T changes, premature ventricular contractions (PVCs) with exercise, left ventricular (LV) chamber size, degree of LV hypertrophy (LVH), New York Heart Association class, LV function, LV fibrosis, LV outflow tract gradient, obstructive sleep apnea, and various HCM genotypes.^6^, ^7^, ^8^, ^9^, ^10^, ^11^, ^12^

LA function and strain mechanics appear to be promising tools for enhanced AF risk stratification and prognostication in HCM. Worsening LA stiffness is associated with significant clinical deterioration and poor outcomes.^13^^,^^14^ HCM patients with AF are at higher risk of stroke, even in the absence of conventional stroke risk factors—namely, congestive heart failure, hypertension, age, diabetes, previous stroke, and vascular disease. Stroke can be the first manifestation of AF in HCM^15^; therefore, risk factor identification before arrhythmia onset is required.

To develop an enhanced AF risk prediction model, clinical and imaging features associated with AF in HCM patients should be recognized. Conventionally, LA function assessment has predominantly relied on echocardiography, which may be suboptimal due to inadequate delineation of atrial borders.^16^ In recent years, cardiac magnetic resonance (CMR) has shown an increased penetrance in clinical practice. CMR has high tomographic spatial resolution with superior characterization of cardiac structures, which can circumvent echocardiography limitations when studying various LA functions and myocardial mechanics.^17^

The purpose of this study was to compare LA phasic function and strain mechanics by CMR between age- and gender-matched HCM patients with vs without AF, to identify novel AF risk markers in HCM. Additionally, we aimed to determine whether an association exists between hemodynamically important LV characteristics and LA remodelling.

## Methods

### Patient selection

Adult HCM patients with CMR imaging who were evaluated in the dedicated HCM clinic at Peter Munk Cardiac Centre, Toronto General Hospital, from January 2001 to June 2021 were included in this retrospective case–control study. The case group was comprised of patients who underwent CMR before the onset of AF.

The diagnosis of HCM was based on the presence of increased LV wall thickness with a nondilated LV chamber in the absence of abnormal cardiac loading conditions. LV outflow tract (LVOT) obstruction was defined by a peak instantaneous outflow gradient of ≥ 30 mm Hg at rest or with physiological exercise.^15^

AF diagnosis was defined by documented AF on an electrocardiogram or Holter monitor (> 30 seconds duration) at the index visit or follow-up visits, or by an established history of paroxysmal or permanent AF. In accordance with American Heart Association/American College of Cardiology/Heart Rhythm Society guidelines, paroxysmal AF included AF that terminated spontaneously or with intervention within 7 days of onset. Permanent AF included AF that was continuous and sustained for > 7 days with a joint clinician–patient decision to not pursue attempts at restoring and/or maintaining sinus rhythm.^18^

HCM patients who had undergone surgical myectomy and developed transient perioperative AF with no recurrence were excluded due to transient surgery-related factors leading to perioperative AF.

Age- and gender-matched HCM patients without an established diagnosis of AF were selected as case controls in a 2:1 fashion. Due to there being more AF events, but fewer eligible non-AF controls, all AF cases with pre-AF CMR and matched controls were included at an approximate 2:1 case–control ratio, consistent with cohort-based feasibility.

This study was approved by the Institutional Research Ethics Board of the University Health Network (Canada), and the need for patient written informed consent was waived for this retrospective study (CAPCR ID = 20-6110).

### CMR acquisition

CMR imaging was performed using a 1.5 or 3.0 Tesla scanner (Siemens, Erlangen, Germany). Images were obtained using steady state, free-procession (SSFP) breath-hold cines in 3 long-axis planes and sequential short-axis slices from the atrioventricular ring to the apex. Gadolinium contrast CMR imaging was performed 10-15 minutes after injection of 0.2 mmol/kg of gadolinium- diethylenetriaminepentaacetate **(**DTPA) with 2-dimensional segmented inversion-recovery. All patients were in normal sinus rhythm at the time of CMR imaging.

### LV volume and mass assessment

For LV assessment, we used commercially available Circle cvi42 software (Circle Cardiovascular Imaging Inc., Calgary, Alberta, Canada). Endo- and epicardial borders were contoured manually in short-axis cine images (SAX) at end-diastole and end-systole. The basal slice was included in the analysis if at least 50% of blood volume was surrounded by myocardium. Papillary muscles were excluded and considered to be part of the blood pool. If available, contour smoothing was applied. Quality control of the contours was performed in the movie mode. Ejection fraction (EF in %) and myocardial mass (Mass in g), end-systolic and end-diastolic volume (ESV and EDV, respectively, in mL) were recorded.

### LA volumes, mechanics, and deformation

**Figure 1:**
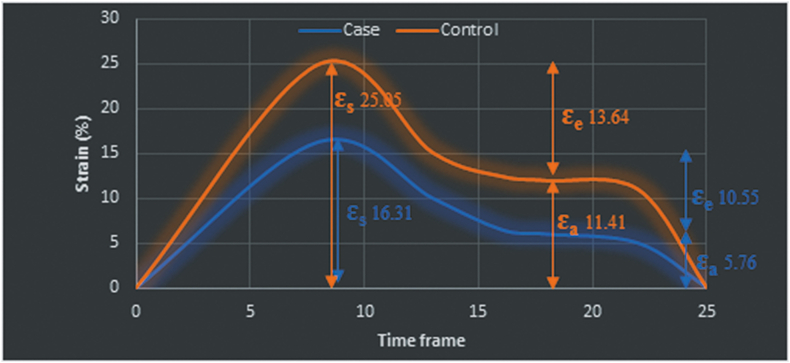
Left atrial strain analysis. Following the R-wave with onset of ventricular systole, there is apical movement of mitral valve apparatus and enlargement of the left atrium corresponding with reservoir strain (Ɛs). In early diastole, there is emptying of the left atrium corresponding with the E-wave and conduit strain (Ɛe). Finally, atrial contraction corresponding to the A-wave is represented as booster strain (Ɛa). As demonstrated, the strain values are lower in patients with hypertrophic cardiomyopathy with atrial fibrillation, compared to those without atrial fibrillation, and this pattern is more prominent in booster function (Ɛa; 5.76 vs 11.41, *P* < 0.001).

LA volume and function were analyzed using commercial postprocessing software (QStrain, Medis Suite 3.2, Leiden, the Netherlands).^19^ The LA endocardial border was manually delineated using a point-and-click approach when the atrium was at its maximum and minimum volumes in both the 2- and 4-chamber cine images (pulmonary veins and LA appendage were excluded; Fig. 1). Then the contours were automatically propagated in all frames throughout the entire cardiac cycle (25 frames/cardiac cycle). CMR-feature tracking was visually reviewed to ensure accurate tracking. In cases of inadequate tracking, the endocardial border was manually readjusted and then the propagation algorithm was reapplied. Tracking was blindly repeated 3 times in both the 2- and 4-chamber views, and the results of the LA volume, strain, and strain rate from the 3 tracking repetitions were averaged in both views. LA strain and strain rate were calculated as the average of the 2- and 4-chamber views.

**Figure 2:**
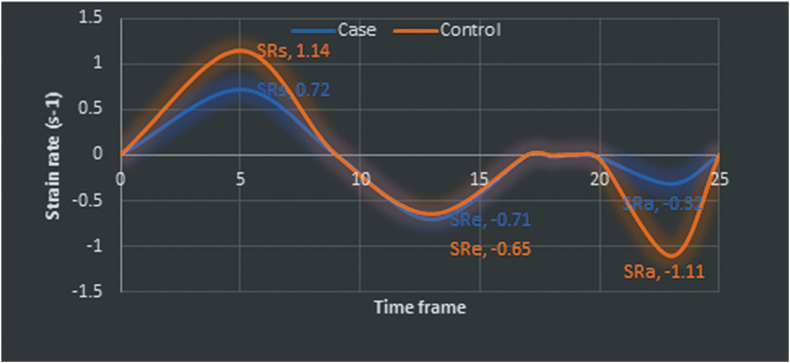
Left atrial (LA) mechanics. Following the R-wave with onset of ventricular systole, there is apical movement of the mitral valve apparatus and enlargement of the left atrium (peak positive wave) corresponding with reservoir strain rate (SRs). In early diastole, there is emptying of the left atrium (peak early negative wave) corresponding with conduit strain rate (SRe). Finally, atrial contraction occurs, represented as a peak late negative wave corresponding with booster strain rate (SRa). The strain rate values are lower in patients with atrial fibrillation. Peak late negative strain rate (SRa), which corresponds to LA booster function, is significantly lower (SRa; –0.32 vs –1.11, *P* < 0.001). However, there is no significant difference in the peak early negative strain rate which corresponds to the LA conduit function (SRe; –0.71 vs –0.65, *P* = 0.418).

Three aspects of LA strain were analyzed (Fig. 2):

1. Total strain (εs, corresponding to LA reservoir function);
2. Active strain (εa, corresponding to LA booster pump function); and
3. Passive strain (εe, corresponding to LA conduit function, the difference between εs and εa).

Accordingly, 3 SR parameters were evaluated (Fig. 2):

1. Peak positive strain rate (SRs, corresponding to LA reservoir function);
2. Peak early negative strain rate (SRe, corresponding to LA conduit function); and
3. Peak late negative strain rate (SRa, corresponding to LA booster pump function).

LA volume (LAV) was assessed at LV end-systole (LAV max), at LV diastole before LA contraction (LAV pre-A), and at late LV diastole after LA contraction (LAV min; Fig. 2). The parameters of the LAV were obtained from a volume curve generated using Simpson’s method. From the LAV, the LA emptying fractions (LAEFs) were calculated as follows:

1. LA total EF = (LAV max – LAV min)/LAV max ∗100%;
2. LA passive EF = (LAV max – LAV pre-A)/LA Vmax ∗100%; and
3. LA active EF = (LAV pre-A – LAV min)/LAV pre-A ∗100%.

### Late gadolinium enhancement (LGE)

CMR images were analyzed for LV mass and LGE with commercially available software (QMASS version 8.0, Medis, Leiden, the Netherlands). LV endocardial and epicardial borders were planimetered manually in cine images on short axis to define the myocardium. Papillary muscles and intertrabecular blood pools were excluded. The LV short-axis stack of LGE images was first assessed visually for the presence of LGE. Quantification of LGE was performed by manually adjusting a grey-scale threshold to define areas of visually apparent LGE. The percentage of LGE was determined as the proportion of LGE relative to the total LV mass.^20^

### Statistical analysis

Continuous variables are presented as mean +/- standard deviation, and categorical variables are presented as frequencies or percentages. Comparisons between patients were performed using the unpaired Student *t* test and the χ^2^ test for continuous and categorical variables, respectively, after testing for normality. The validity and reproducibility were evaluated by intraclass correlations (ICCs). A *P* value < 0.05 was considered statistically significant.

The Kaplan–Meier method was used to estimate AF-free survival from the index CMR date. The relationship between clinical and imaging parameters vs the likelihood of experiencing AF was further evaluated using univariate and multivariable Cox proportional hazards models. The proportional hazards assumption was tested graphically and with time-dependent covariates before proceeding.

Individual parameters with an association with AF were identified by univariate logistic regression analyses. Statistical analysis was performed using SAS for Windows, version 9.3 (SAS Institute, Cary, NC) and MedCalc for Windows, version 15.0 (MedCalc Software, Ostend, Belgium).

## Results

### Demographic and clinical parameters

A total of 977 HCM patients with CMR imaging were evaluated at the HCM clinic at Toronto General Hospital during the defined study period, and AF was identified in 297 (30.4%). The case group comprised of 140 HCM patients (14.3%) with a diagnosis of paroxysmal AF with at least one CMR study before the onset of AF. In the control group, 70 age- and gender-matched HCM patients without any history of AF were randomly selected. Control patients were observed for a median of 60 months (interquartile range, 48-72 months) without any documented AF on serial electrocardiograms, ambulatory monitoring, or review of clinical records. Clinical and baseline characteristics for both subgroups are summarized in Table 1.

**Table 1:** Baseline characteristics of the study population

Characteristic	All patients (n = 210)	Case (n = 140)	Control (n = 70)	*P*
Age, Y	59.88 ± 11.55	60.78 ± 11.92	58.09 ± 10.62	0.111
Sex				0.362
Male	134 (63.8)	86 (61.4)	48 (68.6)	
Female	76 (36.2)	54 (38.6)	22 (31.4)	
BSA, m^2^	1.94 ± 0.25	1.93 ± 0.24	1.95 ± 0.25	0.679
Systemic hypertension	82 (39.1)	57 (40.7)	25 (35.7)	0.376
Diabetes mellitus	28 (13.3)	20 (14.3)	8 (11.4)	0.106
Dyslipidemia	83 (39.5)	57 (40.7)	26 (37.1)	0.275
Obstructive sleep apnea	37 (17.6)	24 (17.2)	13 (18.6)	0.381
Coronary artery disease	23 (10.9)	17 (12.1)	6 (8.6)	0.661
Apical aneurism	4 (1.9)	4 (2.8)	0 (0)	NS
Stroke	3 (1.4)	3 (2.1)	0 (0)	NS
NYHA class				0.37
I/II	189 (90)	122 (87.1)	67 (95.7)	
III	20 (9.5)	17 (12.1)	3 (4.3)	
IV	1 (0.5)	1 (0.7)	0 (0)	
Obstructive HCM	105 (50)	78 (55.7)	27 (38.6)	0.034
Surgical myectomy	29 (13.8)	21 (15)	8 (11.4)	0.389
Medications				
Beta-blocker	152 (72.4)	104 (74.3)	48 (68.5)	0.294
Calcium-channel blocker (dihydropyridine)	14 (12.4)	10 (7.1)	4 (5.7)	0.517
Disopyramide	18 (8.6)	13 (9.3)	5 (7.1)	0.138
Statin	76 (36.2)	53 (37.8)	23 (32.8)	0.412
ACEI / ARB	51 (24.3)	39 (27.8)	12 (17.1)	0.207
Genetic test				NS
Pathogenic	34 (16.3)	26 (18.6)	8 (11.4)	
Negative	99 (47.1)	62 (44.3)	37 (52.9)	
VUS	32 (15.2)	13 (9.3)	19 (27.1)	
Unknown or N/A	45 (21.4)	39 (27.8)	6 (8.6)	

Data are presented as mean ± standard deviation, or frequency (proportion). ACEI, angiotensin-converting enzyme inhibitor; ARB, angiotensin receptor blocker; BSA, body surface area; HCM, hypertrophic cardiomyopathy; N/A, not applicable; NS, not significant; NYHA, New York Heart Association; VUS, variant of unknown significance.

The mean age of the study cohort at the time of CMR was 58.9 ± 11.6 years (range, 25-87). Most patients (n = 189; 90%) were New York Heart Association class I/II and were on beta-blockers (n = 152; 72.4%). Medication use was similar in the 2 groups. No significant differences were present in body surface area, comorbidity profile (including hypertension, diabetes mellitus, dyslipidemia, and obstructive sleep apnea), or coronary artery disease history.

LVOT obstruction was significantly more prevalent in the AF group; however, the invasive septal reduction procedure rate was similar between the 2 groups. Three stroke events and 4 LV apical aneurysms occurred in our study cohort, all in the AF group. No significant difference was present in genetic testing results between the 2 groups.

### Phasic LA volumes, function, and LA mechanics

Patients with AF had larger LA volumes and worse atrial function than those with sinus rhythm. Specifically, absolute LA maximum, pre-A, and minimum volumes were all significantly higher in the AF group, and these differences remained significant after indexation to body surface area. Additionally, total, passive, and active LA emptying fractions were significantly lower in AF patients compared with those in controls (Table 2).

**Table 2:** Left atrial (LA) and left ventricular (LV) characteristics by cardiac magnetic resonance (CMR) imaging

LA volume and mechanics by CMR	Case (n = 140)	Control (n = 70)	*P*
**LA volumes, mL**			
Maximum	116.55 ± 41.42	89.41 ± 34.95	< 0.000
Pre-A	92.23 ± 37.17	66.61 ± 33.23	< 0.000
Minimum	80.07 ± 35.72	53.31 ± 33.07	< 0.000
Maximum	116.55 ± 41.42	89.41 ± 34.95	< 0.000
**LAEF, %**			
Total	32.85 ± 12.75	44.01 ± 9.29	< 0.000
Passive (Conduit)	21.59 ± 10.48	27.74 ± 8.63	< 0.000
Active (Booster)	14.45 ± 7.52	22.11 ± 8.72	< 0.000
**LA strain, ϵ, %**			
**ϵ_s_** (Reservoir)	16.31 ± 7.46	25.05 ± 7.05	< 0.000
**ϵ_e_** (Conduit)	10.55 ± 5.94	13.64 ± 5.54	< 0.000
**ϵ_a_** (Booster)	5.76 ± 3.35	11.41 ± 4.17	< 0.000
**ϵ_a_/ϵ_s_** (Booster/reservoir)	0.36 ± 0.15	0.46 ± 0.14	< 0.000
**LA strain rate, SR, s^–1^**			
SRs	0.72 ± 0.31	1.14 ± 0.35	< 0.000
SRe	–0.71 ± 0.45	–0.65 ± 0.28	0.418
SRa	–0.32 ± 0.31	–1.11 ± 0.43	< 0.000

LV characteristics by CMR	Case (n = 140)	Control (n = 70)	*P*
Maximum wall thickness, mm	18.93 ± 4.09	17.39 ± 3.24	0.007
mass, g	166.07 ± 63.15	148.31 ± 50.65	0.044
Mass index, g/m^2^	86.13 ± 28.94	75.12 ± 21.76	0.006
Ejection fraction, %	61.88 ± 8.61	62.88 ± 6.06	0.387
End diastolic volume index, mL/m^2^	85.85 ± 22.83	78.47 ± 12.81	0.013
End systolic volume index, mL/m^2^	33.69 ± 15.65	29.25 ± 6.99	0.050
**Evidence of LGE on contrast-enhanced CMR, n (%)**	134	70	0.135
No evidence or minimal	54 (40.3)	28 (40)	
Mild to moderate (5%–15% LV mass)	63 (47)	39 (55.7)	
Moderate to severe (> 15% LV mass)	17 (12.7)	3 (4.3)	

LAEF, left atrium ejection fraction; LGE, late gadolinium enhancement; ϵ, left atrial strain; εs, total strain; ϵ_e,_ passive strain; ϵ_a,_ active strain; SR, strain rate; s^-1^, the inverse value of a second; SRa, peak late negative strain rate;.SRe, peak early negative strain rate; SRs, peak positive strain rate.

There was a significant difference in LA strain and strain rate parameters between HCM patients with AF vs without AF. Patients with AF had lower total (reservoir), passive (conduit), and active (booster) strain than did patients without AF.

Similarly, peak positive strain rate and peak late negative strain rate were lower in AF patients compared to non-AF patients, whereas the peak early negative strain rate did not differ significantly between the 2 groups (Table 2).

### LV parameters

Maximum LV wall thickness, absolute and indexed LV mass, and LV end diastolic volume index were all higher in patients with AF compared to the non-AF group.

LVEF by CMR as well as prevalence and pattern of LGE, were similar in the 2 groups, although a higher proportion of patients with AF exhibited severe LGE (Table 2).

### Time-to-event analysis

By univariate Cox regression analysis, a significant relationship was present between development of AF and LA maximum, LA pre-A, LA minimum volumes, total LAEF, passive LAEF, active LAEF, LA total strain, LA passive strain, LA active strain, peak positive strain rate, and peak late negative strain rate (all *P* < 0.05; Table 3).

**Table 3:** Univariate regression analysis

Left atrial measures	Unit	Odds ratio	95% CI	*P*
**Volumes, mL**				
Maximum	5	1.357	1.208–1.525	< 0.0001
Pre-A	5	1.477	1.281–1.703	< 0.0001
Minimum	5	1.643	1.382–1.954	< 0.0001
**Emptying fraction, %**				
Total	5	0.644	0.550–0.755	< 0.0001
Passive (Conduit)	5	0.734	0.630–0.856	< 0.0001
Active (Booster)	5	0.558	0.452–0.689	< 0.0001
**Strain, ϵ, %**				
ϵ_s_ (Reservoir)	5	0.452	0.352–0.582	< 0.0001
ϵ_e_ (Conduit)	5	0.643	0.499–0.828	< 0.0001
ϵ_a_ (Booster)	1	0.687	0.619–0.763	< 0.0001
**SR, s^–1^**				
SRs	0.1	0.662	0.582–0.752	< 0.0001
SRe	0.1	0.971	0.905–1.042	0.281
SRa	0.1	0.612	0.426–0.822	< 0.0001

CI, confidence interval; pre-A, LA volume assessed at LV diastole before LA contraction; ϵ, left atrial strain; ε_s_, total strain; ϵ_e,_ passive strain; ϵ_a,_ active strain; SR, strain rate; s^-1^, the inverse value of a second; SRa, peak late negative strain rate; SRe, peak early negative strain rate; SRs, peak positive strain rate.

**Figure 3:**
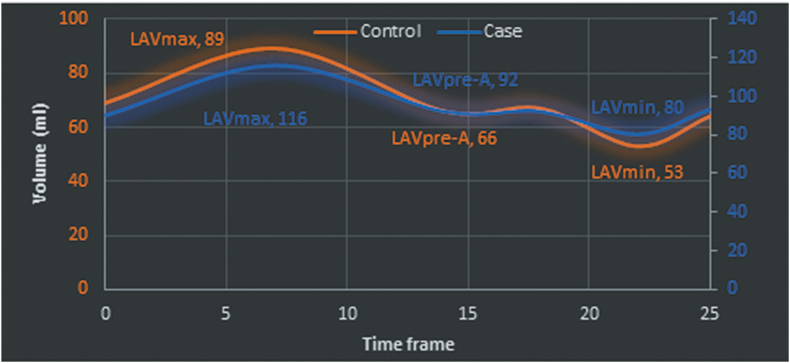
A time-volume curve; LAVmax, maximal left atrial (LA) volume at left ventricular (LV) end-systole; LAV pre-A is the LA volume at LV diastole before LA contraction; LAV minimum is the minimal LA volume at LV end-diastole, demonstrating higher volumes in all 3 phases of LA function in patients with hypertrophic cardiomyopathy with atrial fibrillation. Although the volumes appear to rise and fall in parallel, the differences in strain and strain rate are better demonstrated in Figure 2, due to differences in starting and emptying volumes.

**Figure 4:**
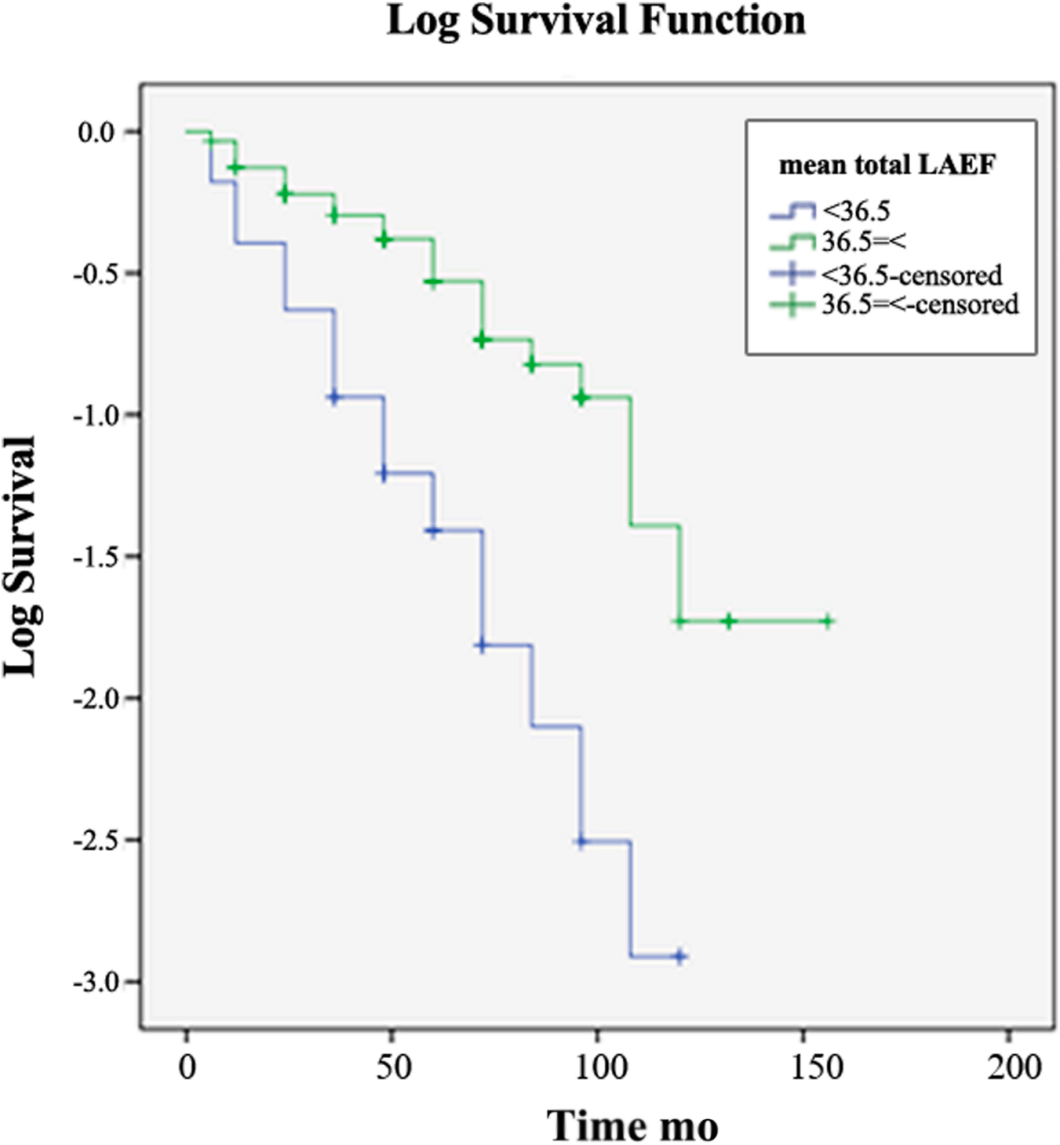
Kaplan–Meier curve demonstrating time to event for patients with hypertrophic cardiomyopathy using total left atrial ejection fraction (LAEF). The multivariate logistic regression showed that the lower the left atrial ejection fraction, the greater the possibility of developing atrial fibrillation in the future. The LAEF cutoff for developing atrial fibrillation in this study was 36.5%.

**Figure 5:**
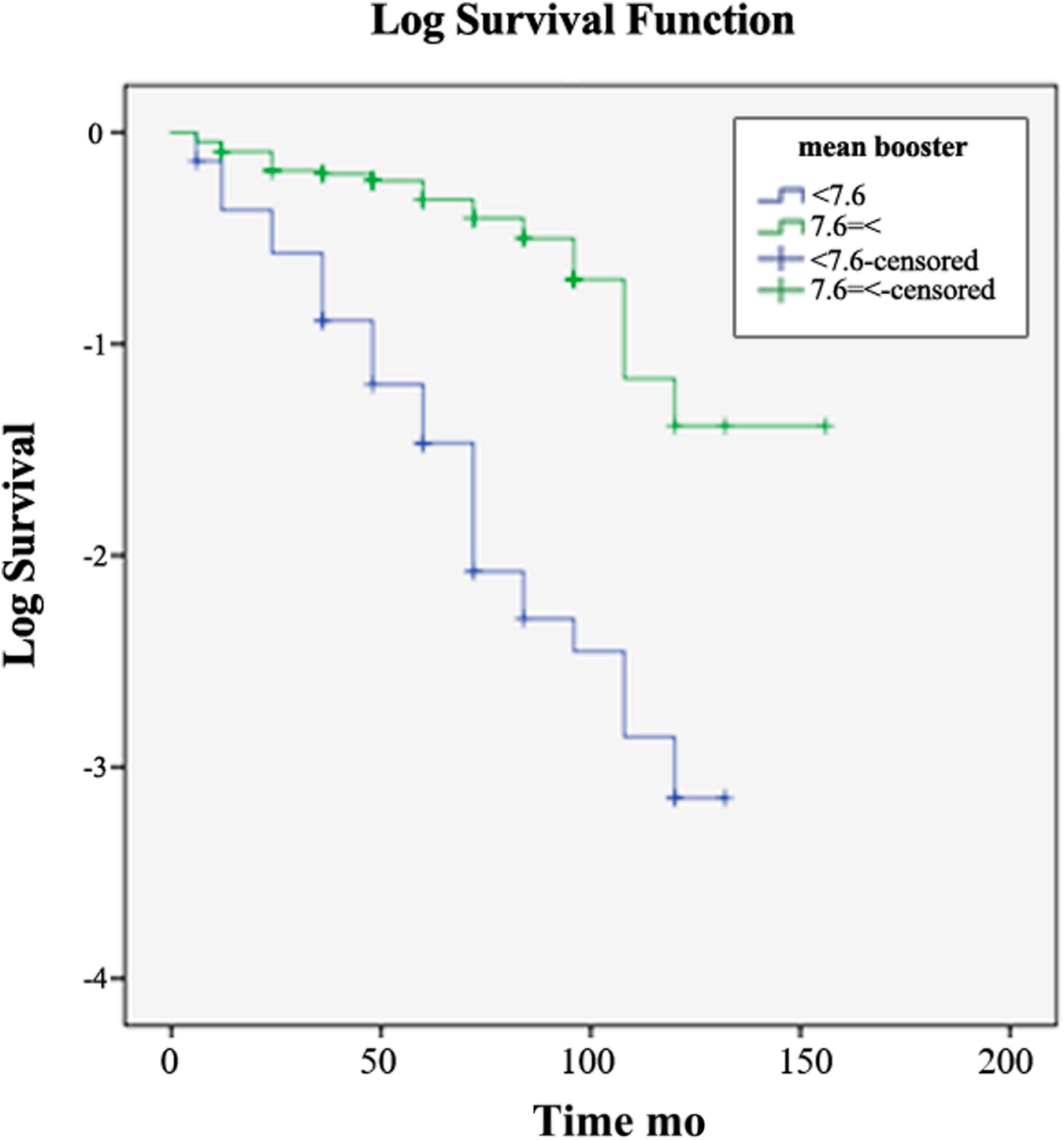
Kaplan–Meier curve demonstrating time to event for patients with hypertrophic cardiomyopathy using left atrial (LA) booster strain. The multivariate logistic regression showed that the lower the LA booster strain, the greater the possibility of developing atrial fibrillation in the future. The LA booster strain cutoff for developing atrial fibrillation in this study was 7.6%.

However, using stepwise multivariable regression analyses, only LA active strain (booster function) and total LAEF remained independently associated with AF (Table 4; Fig. 3). The Kaplan-Meier survival curves demonstrating time to event based on total LAEF and LA booster strain cutoffs are shown in Figure 4, Figure 5, respectively

**Table 4:** Final multivariable analysis with adjusted analysis for unadjusted confounders

	HR	95% HR CI	*P*
Final multivariable analysis			
Booster	0.935	0.882–0.991	0.024
Total LAEF	0.979	0.962–0.995	0.012
LA Booster strain and total LAEF with: HTN, DM, age			
Booster	0.933	0.880–0.990	0.022
Total_LAEF	0.978	0.961–0.995	0.012
HTN	0.814	0.562–1.178	0.276
DM	0.885	0.536–1.461	0.633
Age	1.009	0.993–1.025	0.294
LA Booster strain and total LAEF with: HTN, DM, age, and sex			
Booster	0.934	0.880–0.991	0.025
Total_LAEF	0.978	0.961–0.995	0.013
Sex	0.972	0.676–1.396	0.876
HTN	0.814	0.563–1.179	0.277
DM	0.884	0.535–1.459	0.629
Age	1.008	0.992–1.025	0.311

CI, confidence interval; DM, diabetes mellitus; LA, left atrial; LAEF, LA ejection fraction; HR, hazard ratio; HTN, hypertension.

In the multivariate regression analysis, done by incorporating the patient’s age at the time of CMR, LV maximum wall thickness and LV mass index variables were not shown to be associated with development of AF.

## Discussion

LA remodelling has been demonstrated to have a primary role in the pathogenesis of AF. Among all features of LA remodelling, LA enlargement in the setting of volume or pressure overload has the clearest connection.^21^ Diastolic dysfunction secondary to LV hypertrophy/fibrosis or LVOT obstruction, which are seen in the majority of HCM patients, can lead to chronically elevated filling pressures, and in turn, LA pressure overload.^22^

LA size is an independent marker of HCM severity and is connected to its prognosis.^23^ However, an increasing pool of data suggests that hemodynamic factors are not the only players in this field, and that atrial cardiomyopathy could in fact play an important role. Eshoo et al. have shown that the patients with HCM have larger LA volumes and poorer function, compared to those of patients with hypertension and comparable LV mass.^24^ Additionally, in a significant subset of HCM patients, AF occurs at a relatively earlier age than expected, and this arises from LA remodelling rather than hemodynamic stresses alone.^10^ This premise is further supported by the lower success rates of AF ablation in patients with HCM, compared to those in non-HCM patients.^25^

Indeed, prior studies have sought to examine the relationship between LA size and function with the occurrence of AF in HCM patients, with the aim of determining predictive factors. Like non-HCM patients, HCM patients with AF have universally increased LA size, whether assessed by A-P diameter, area, or maximum and minimum volumes.^26^ In a large study by Maron et al. consisting of 427 HCM patients, total LAEF < 38%, LA end diastolic volume > 118 mL, and age ≥ 40 years were all found to be independently associated with AF occurrence.^21^

LA function, however, is more complex and is characterized by 3 distinctive functions, which enable modulation of LV filling, including LA reservoir, conduit, and booster functions.^27^ In this study, we examined the role of LA volumes, all LA phasic functions, and myocardial deformation to predict the development of AF over time.

Our study has several novel findings that provide further insight into LA pathophysiology in HCM patients with AF. Consistent with previous studies, we demonstrated that HCM patients with AF had larger LA volumes (maximum, pre-A, and minimum) and reduced LA emptying fractions (total, passive, and active). We also found concomitant reductions in LA strain (total, passive, and active) and LA strain rate parameters. Among HCM patients who developed AF, decreased LA function is more prominent in terms of booster function, compared with reservoir or conduit function. In HCM patients, all 3 components of LA mechanics (strain analysis) were reduced, but the degree of reduction was more prominent in the booster function in the AF group compared to that in the non-AF group.

Although prior studies have shown that HCM patients in general have reduced LA myocardial deformation, only limited data are available comparing phasic LA strain in HCM patients with vs without AF.^28^ Evaluating LA function by myocardial deformation has the advantage of directly assessing endocardial contraction, independent of LA volume measurements, which assume LA structure to be geometrically symmetric and spherical.^19^

Debonnaire et al. showed the utility of LA strain as a predictor of AF in a cohort of 242 HCM patients.^29^ A study by Raman et al., like ours, showed in a group of 33 HCM patients who developed AF, that age > 55 years and LA booster function by strain < 8% were components augmenting the AF prediction model in HCM.^30^ We have shown in our study that a lower LA booster function (by strain or strain rate) portends a higher risk for developing AF.

In our cohort, patients with HCM who developed AF had markedly reduced LA booster strain (5.8% ± 3.4%), compared with those who remained in sinus rhythm (11.4% ± 4.2%), and LA booster strain emerged, together with total LAEF, as an independent predictor of AF in multivariable analysis. The level of impairment we observed is very similar to that reported in previous CMR studies of HCM, in which low booster strain has consistently identified patients at higher risk of AF, supporting LA active strain as a clinically meaningful marker of atrial myopathy and AF risk in this population.^30^^,^^31^

We also investigated whether any LV measures were associated with AF in HCM patients. Maron et al. have previously shown no meaningful association with LV mass, maximum LV wall thickness, LVEF, LV end diastolic volume, or extent of LGE,^21^ although some smaller studies have suggested that degree of LGE is correlated with AF development.^7^^,^^32^ In our study, we showed comparable LVEF and LGE burden between HCM patients with vs without AF. We did observe overall greater LV maximum wall thickness, LV mass, LV end diastolic volume, and slightly lower LVEF in our AF group, but these differences were not statistically significant in the multivariable analysis.

Mechanistically, the changes observed in LA function associated with AF suggest not only impairment in atrial compliance, but also, more significantly, in atrial contraction. The loss in capacity for the left atrium to act as a reservoir and accommodate blood volume that can be transmitted to the left ventricle, coupled with a reduced ability to contract, suggests that the left atrium functions more as a passive conduit.

Indeed, our multivariable model, which included both LA and LV parameters, shows that LA active strain (booster function) correlates independently with AF in HCM patients and in fact improves the predictive model for AF when added to LA volumetric assessment parameters (total LAEF).

Although there is consensus that LA volume and LAEF offer adequate information for AF risk assessment, our data show significant overlap in these measures between patients with vs without AF. Given this overlap, LA mechanics analysis, especially LA booster function assessment can potentially provide clinicians with another tool in their armamentarium to help predict which HCM patients may be at most risk of developing AF and thus requiring closer surveillance.

### Limitations

This retrospective study was conducted with data from a single HCM centre. HCM patients with AF were included only if they had CMR imaging before the AF event. Although the study may carry potential for patient selection bias and limits to generalizability, HCM patients seen at our centre are routinely sent for CMR imaging unless contraindications are present, such as presence of an intracardiac defibrillator. In addition, we did not assess or compare the patients with CMR after their AF event. We also did not compare this model to the other clinical models, such as HCM-AF, Coronary artery disease/dysfunction, Hypertension, Elderly age ≥75, Systolic dysfunction, Thyroid disease (C2HEST), **C**ongestive Heart Failure, **H**ypertension, **A**ge ≥ 75 years, **D**iabetes, **S**troke/Transient Ischemic Attack, **V**ascular Diseases, **A**ge 65 to 74 years, **S**ex **C**ategory (CHA_2_DS_2_VASc), and Cohorts for Heart and Aging Research in Genomic Epidemiology–Atrial Fibrillation (CHARGE-AF). We also did not compare echocardiography parameters with CMR. Prospective studies that combine same-time echocardiography and CMR, with full diastolic assessment and echo-derived LA strain, are important to show how CMR-based LA strain adds to and complements echocardiographic measures in defining diastolic burden and AF risk in HCM. Eventually, LVOT obstruction was assessed just as a categorical variable (obstructive vs nonobstructive), and standardized resting/dynamic LVOT gradients at the time of CMR were not consistently available.

## Conclusion

LA remodelling is associated with development of AF in HCM patients. This likely occurs in the setting of an atrial myopathy which is, at least, in part independent of changes in response to hemodynamic factors related to LV burden of disease. Although LA volume and LAEF are important risk predictors for AF, significant overlap between patients with vs without AF may limit their use.

We demonstrated that LA strain analysis with CMR, specifically booster function, could augment the prediction tools for AF and subsequently for cerebrovascular events in HCM patients.

## Ethics Statement

This study was approved by the Institutional Research Ethics Board of the University Health Network (Canada). The research reported in this paper adhered to the Declaration of Helsinki guidelines.

## Patient Consent

The authors confirm that patient consent was not required for this article, as it is based on a retrospective review of de-identified data and was exempt from institutional review board consent requirements.

## Funding Sources

The authors have no funding sources to declare.

## Disclosures

The authors have no conflicts of interest to disclose.
